# Assessing the eligibility of Brainomix e-ASPECTS for acute stroke imaging

**DOI:** 10.3389/fninf.2025.1668395

**Published:** 2025-12-11

**Authors:** Mateusz Dorochowicz, Arkadiusz Kacała, Michał Puła, Adrian Korbecki, Aleksandra Kosikowska, Aleksandra Tołkacz, Anna Zimny, Maciej Guziński

**Affiliations:** 1 Department of General, Interventional and Neuroradiology, Wroclaw University Hospital, Wrocław, Poland; 2 Department of Radiology, Wroclaw Medical University, Wroclaw, Poland; 3 Department of General, Interventional and Neuroradiology, Wroclaw Medical University, Wrocław, Poland; 4 Faculty of Medicine, Wroclaw Medical University, Wrocław, Poland

**Keywords:** e-ASPECTS, acute ischemic stroke, artificial intelligence in stroke imaging, mechanical thrombectomy eligibility, clinical decision support systems (CDS system), stroke imaging algorithms, healthcare AI validation

## Abstract

**Background:**

Timely and accurate assessment of acute ischemic stroke is crucial for determining eligibility for mechanical thrombectomy. The Alberta Stroke Program Early CT Score (ASPECTS) is a widely used tool for evaluating early ischemic changes on non-contrast CT (NCCT), but its interpretation is subject to interobserver variability. Brainomix e-ASPECTS is an automated software designed to standardize and expedite this assessment. We aimed to evaluate the clinical utility and diagnostic performance of the Brainomix e-ASPECTS software in an unselected, real-world cohort of patients undergoing NCCT for suspected acute ischemic stroke.

**Methods:**

We retrospectively analyzed 1,029 NCCT studies from 954 patients between March 2020 and December 2024. e-ASPECTS scores were compared to radiologist-assigned ASPECTS, which served as the reference standard. Diagnostic accuracy, sensitivity, specificity, and correlation between scoring methods were assessed.

**Results:**

There was a strong correlation between e-ASPECTS and radiologist ASPECTS (*ρ* = 0.953, *p* < 0.001). For detecting acute ischemia, sensitivity was 95.8% (95% CI, 93.6–97.3%), specificity 96.9% (95% CI, 94.7–98.2%), and overall accuracy 96.3% (95% CI, 95.1–97.5%). The positive predictive value was 97.2% (95% CI, 95.3–98.4%), and the negative predictive value was 95.3% (95% CI, 92.8–96.9%). Score concordance was high, with exact matches in 92.3% of cases and a ≤ 1-point difference in 97.7%. Misclassification for thrombectomy eligibility (ASPECTS < 6) occurred in four cases (0.4%). The software achieved a processing success rate of 91.9%.

**Conclusion:**

E-ASPECTS demonstrates high diagnostic accuracy and strong agreement with expert radiological assessment, supporting its role as a valuable decision support tool in acute stroke imaging. However, its use should complement, not replace, expert interpretation, particularly in patients with low ASPECTS scores, where treatment decisions are most sensitive.

## Introduction

In acute stroke imaging, time is a critical factor in determining eligibility for mechanical thrombectomy, with faster decision-making directly linked with the shorter time to recanalisation, which in turn leads to improved clinical outcomes (Heit et al., 2021; Alawieh et al., 2019; Yang et al., 2022). Therefore, every method that can accelerate and simplify the assessment process, without compromising diagnostic accuracy, is invaluable. The selection of patients for mechanical thrombectomy in cases of acute ischemic stroke depends significantly on imaging criteria, especially the Alberta Stroke Program Early CT Score (ASPECTS).

ASPECTS is a 10-point semiquantitative system that has helped standardize the evaluation of early ischemic changes on NCCT in patients with acute ischemic stroke affecting the middle cerebral artery (MCA) territory. Starting from a maximum score of 10, one point is deducted for each ASPECTS region showing signs of ischemia, with lower scores indicating larger infarct volumes. The clinical utility of ASPECTS extends beyond diagnostic standardization, as it plays a pivotal role in patient selection for recanalization therapies, including intravenous thrombolysis and mechanical thrombectomy. Numerous studies have demonstrated that higher ASPECTS values are strongly associated with improved functional outcomes following both interventions, whereas lower scores correlate with larger infarct cores and an increased risk of hemorrhagic transformation (Barber et al., 2000; Naylor et al., 2017).

Early thrombectomy trials set the foundation for imaging-based selection by excluding patients with large infarcts. Protocols such as ESCAPE and REVASCAT collectively established ASPECTS ≥6 as a practical threshold in clinical guidelines to avoid treating patients with extensive ischemic injury (Goyal et al., 2015; Jovin et al., 2015). However, more recent large randomized controlled trials, including SELECT2, ANGEL-ASPECT, and RESCUE-Japan LIMIT, have demonstrated that patients with ASPECTS as low as 3–5 may still derive significant benefit from thrombectomy (Sarraj et al., 2023; Huo et al., 2023; Yoshimura et al., 2022). These evolving data highlight the need for precise and reproducible ASPECTS assessment, especially in patients with low scores, where treatment decisions are most sensitive.

Although ASPECTS has become a key imaging criterion for selecting candidates for mechanical thrombectomy in cases of large vessel occlusion, it is not without limitations. The detection of early ischemic changes on NCCT can be challenging due to the subtlety of findings such as parenchymal hypoattenuation and loss of gray-white matter differentiation. These subtle changes may be difficult to discern, even for experienced clinicians, potentially leading to underestimation or overestimation of infarct size (Mueller et al., 2022; Bal et al., 2015). Some studies report that the sensitivity of NCCT for detecting ischemia within the first 3–6 h after onset ranges from 43 to 71%, while others have shown that even with optimized ‘stroke window’ settings, detection rates may reach up to 70% (Samaniego et al., 2023; Mainali et al., 2014). ASPECTS scoring is subject to interobserver and intra-observer variability. Studies have reported moderate agreement levels among different readers, with variability influenced by factors such as reader experience, assessed region, and image quality (Farzin et al., 2016; Nicholson et al., 2020).

The development of automated algorithms capable of accurately identifying infarcts within the ASPECTS framework is essential to enhancing consistency and reliability in stroke imaging assessment. By mitigating subjectivity and minimizing reader-dependent variability, such tools have the potential to standardize ASPECTS scoring across clinical settings, leading to more uniform patient selection for mechanical thrombectomy. Moreover, automated analysis has the potential to substantially reduce interpretation time when used as a supportive tool to augment radiologist performance and decision-making.

Independent external validation of clinical decision support tools, such as Brainomix (Oxford, UK) e-ASPECTS, is essential to ensure their reliability and generalizability across diverse clinical settings (Mallon et al., 2024; Yu et al., 2022). While initial studies often utilize controlled datasets with selected patient populations, real-world clinical environments present a broader spectrum of cases, including patients with prior strokes, imaging artifacts, or stroke mimics (Brinjikji et al., 2021; Neuhaus et al., 2020; Austein et al., 2019; Goebel et al., 2018). Validating e-ASPECTS in these heterogeneous populations is crucial to assess its performance and utility in routine practice.

In this study, we conducted a retrospective evaluation of the Brainomix e-Stroke e-ASPECTS software in a consecutive cohort of patients presenting with suspected acute ischemic stroke. The cohort was unselected in the sense that it included all consecutive cases referred for NCCT assessment in routine clinical practice, with exclusions limited to quality-based factors such as motion artifacts, incomplete imaging, hemorrhage, or intracranial tumors. The analysis focused on assessing the software’s utility as a decision-support tool for patient selection in the context of mechanical thrombectomy.

## Methods

### Patients/cohort

In this retrospective study, we conducted a thorough evaluation of a consecutive cohort of patients with suspected acute stroke between March 2020 and December 2024, who underwent an NCCT of the head and whose imaging was analyzed by Brainomix e-Stroke software. Patients were excluded from the analysis if NCCT revealed intracranial mass lesions (including primary neoplasms or metastases) or haemorrhagic stroke (since e-ASPECTS suppresses information related to possible ischemia if it detects a hyperdense region, such as acute haemorrhage). If a patient was admitted on multiple occasions, each admission was treated as a separate study; however, repeat cases involving the same patient were documented accordingly, and their prior clinical history and imaging were taken into consideration during assessment. No clustering adjustment was applied, as repeat admissions represented only a small proportion of the dataset.

Patients were scanned using two CT systems. Examinations were performed either on a Revolution Apex 256-row scanner (GE Healthcare, Chicago, IL, USA) with an axial acquisition protocol or on a LightSpeed VCT 64-row scanner (GE Healthcare). For the Revolution Apex, parameters included: supine positioning with the head fixed in a dedicated holder, tube voltage 120 kVp, tube current 320 mA, beam collimation 80 mm, slice thickness 0.625 mm, coverage from the C1 vertebral body to the vertex, rotation time 1 s, and activation of automatic exposure control (AEC). For the LightSpeed VCT, acquisition settings were matched to those of the Revolution Apex, except for beam collimation (2.5 mm for the infratentorial region and 5 mm for the supratentorial region) and a single gantry rotation coverage of 40 mm. The scan range extended from the foramen magnum to the top of the head.

Imaging data were retrieved from the PACS system, and automated e-Stroke results were obtained from the corresponding DICOM-based outputs. Clinical data were extracted from a prospectively maintained database of patients admitted with suspected acute stroke.

### e-ASPECTS

All patients with suspected acute stroke were sent to the Brainomix cloud-based servers for analysis. e-ASPECTS scoring was performed using standard operating procedures, in accordance with previously established methodology (Nagel et al., 2017; Nagel et al., 2018; Pfaff et al., 2017). Following pre-processing of pseudonymized DICOM input images, a registration step was performed to correct for any 3D tilt, rotation, or other positional variations. Image features were then extracted and analyzed using a machine learning–based classifier to identify early ischemic changes. ASPECTS regions were automatically segmented and classified as either ischemic or normal-appearing. Regions identified as non-acute hypodensity were treated as unaffected for the purpose of this analysis. The result of e-ASPECTS was automatically sent to PACS in DICOM format.

### Manual ASPECTS evaluation

ASPECTS scoring was performed on the NCCT images by radiologists assigned to emergency case evaluation, all of whom were trained in the appropriate application of the ASPECTS methodology. Given that the radiologists’ assessments were considered the reference standard, readers evaluated all available image slices rather than restricting analysis to a single supraganglionic and ganglionic level as outlined in the original ASPECTS methodology. They were also allowed to review prior imaging when available, adjust window and level settings at their discretion, and incorporate relevant clinical information provided at the time of assessment. Radiologist-assigned ASPECTS was used as the reference standard, as the study aimed to assess e-ASPECTS performance as a clinical decision-support tool rather than as a surrogate for MRI-based infarct volume.

### Statistical analysis

Continuous variables such as e-ASPECTS score and ASPECTS score are presented as median with interquartile range (IQR). Categorical variables such as the presence of acute ischemia and misclassification for thrombectomy eligibility are summarized as frequencies and percentages. Diagnostic performance of Brainomix in detecting ischemic change was evaluated against radiologist-assigned ASPECTS (reference standard). Sensitivity, specificity, accuracy, predictive values, and likelihood ratios were calculated together with their 95% confidence intervals (CIs). CIs were estimated using the Wilson method. To assess concordance between e-ASPECTS and radiologist-assigned ASPECTS scores, exact agreement was calculated as the proportion of identical scores, with 95% confidence intervals (CIs) estimated using the Wilson method. Agreement was further stratified into clinically relevant categories (ASPECTS <6 vs. ≥6), reflecting thresholds commonly applied for thrombectomy eligibility. Cohen’s kappa statistic (unweighted) was also computed to quantify agreement beyond chance. Linear relationships between variables were assessed using Pearson’s correlation coefficient. A two-tailed *p*-value of <0.05 was considered statistically significant. All statistical analyses were performed using R version 4.5. 0 (R Foundation for Statistical Computing, Vienna, Austria) (R Core Team, 2021).

## Results

### Patient population/cohort

**Figure 1:**
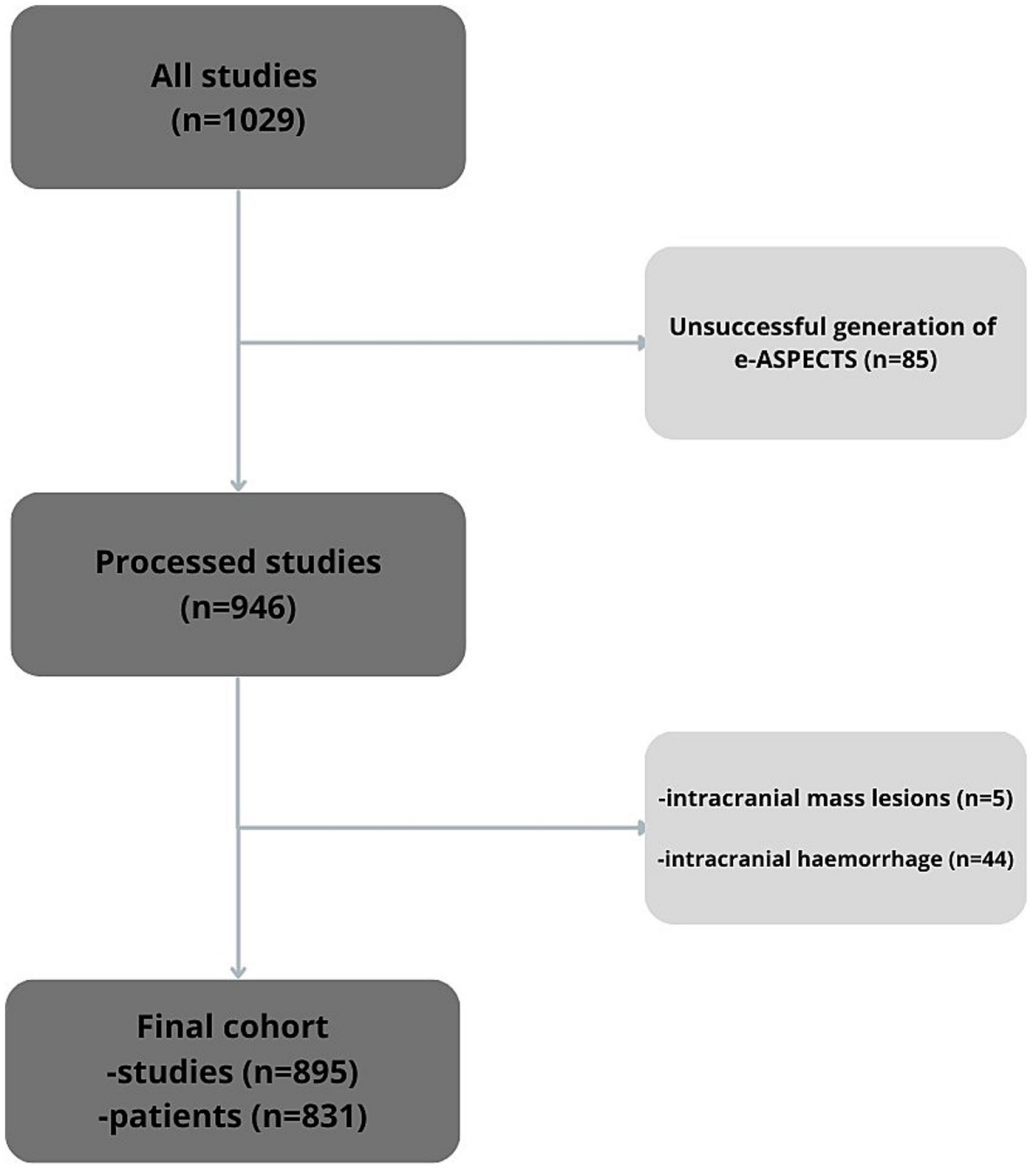
Flow chart of the study cohort.

A total of 1,029 imaging studies from 954 patients who underwent NCCT for suspected acute ischemic stroke were retrospectively reviewed. Of these, 85 cases were excluded due to unsuccessful generation of e-ASPECTS scores, primarily due to significant motion artifacts, with a small number of scans failing for undetermined technical reasons (e.g. truncated image ranges or incomplete series). An additional 5 cases were excluded due to the presence of intracranial mass lesions, and 44 cases were excluded following the identification of intracranial haemorrhage. After applying these exclusion criteria, a total of 895 imaging studies from 831 unique patients were included in the final analysis. Figure 1 shows a flow chart of the study cohort.

A heatmap comparing e-ASPECTS and ASPECTS scores is presented in Table 1. For patients with an acute infarct, the median score was 8 for both e-ASPECTS and ASPECTS. There was a strong positive correlation between the two scoring methods (*ρ* = 0.953, *p* < 0.001).

**Table 1:** A heatmap comparing e-ASPECTS and ASPECTS scores.

e-ASPECTS	10	0	0	0	0	0	0	0	0	3	10	457
	9	0	0	0	0	0	1	1	2	11	172	14
	8	0	0	0	0	1	0	2	3	99	2	2
	7	0	0	0	0	0	0	3	51	1	1	4
	6	0	0	0	0	0	0	19	2	1	0	0
	5	0	0	0	0	0	11	0	1	0	0	0
	4	0	0	0	0	10	1	0	1	0	0	0
	3	0	0	0	2	0	1	0	0	0	0	0
	2	0	0	3	0	0	0	0	0	0	0	0
	1	0	3	0	0	0	0	0	0	0	0	0
	0	0	0	0	0	0	0	0	0	0	0	0
		0	1	2	3	4	5	6	7	8	9	10
		ASPECTS										

Table is presented as a heatmap, where the color shading reflects the frequency of each specific ASPECTS and e-ASPECTS score combination. Sequential scale visually represents the intensity of the data, allowing for a quick identification of the pattern.

The diagnostic performance of e-ASPECTS in the detection of ischemia was high. Sensitivity was 95.8% (95% CI, 93.6–97.3%), specificity 96.9% (95% CI, 94.7–98.2%), and overall accuracy 96.3% (95% CI, 95.1–97.5%). The positive predictive value was 97.2% (95% CI, 95.3–98.4%), and the negative predictive value was 95.3% (95% CI, 92.8–96.9%). Stratified analysis showed that agreement between e-ASPECTS and radiologist scoring was high across both clinical strata. In the ASPECTS <6 group (*n* = 33), exact agreement was 87.9% (95% CI: 72.7–95.2) with a Cohen’s kappa of 0.83. For patients with ASPECTS ≥6 (*n* = 862), agreement was even higher at 92.6% (95% CI: 90.6–94.1) with a kappa of 0.88. Considering the full cohort (*n* = 895), overall agreement reached 92.4% (95% CI: 90.5–94.0), corresponding to a kappa of 0.88, indicating substantial concordance between automated and expert ASPECTS assessment. Table 2 shows the performance of e-ASPECTS in the detection of acute infarcts. The e-ASPECTS and traditional ASPECTS scores were exactly the same in 827 (92.3%) cases, and differed by no more than one point in 875 cases (97.7%). Among those with confirmed acute infarction, both scoring methods gave identical results in 370 cases (88.3%).

**Table 2:** The performance of e-ASPECTS in the detection of acute infarcts (APSECTS < 10).

	Radiologist		
Brainomix		Ischemic change	No ischemic change
	Ischemic change	True positive = 405	False positive = 20
	No ischemic change	False negative = 13	True negative = 457

Using an ASPECTS threshold of less than 6 to determine eligibility for mechanical thrombectomy, reliance on e-ASPECTS alone would have led to misclassification in four patients: two would have been incorrectly excluded from treatment, and two would have been incorrectly considered eligible.

## Discussion

This study aimed to evaluate the clinical utility and diagnostic performance of the Brainomix e-ASPECTS software in an unselected cohort of patients undergoing NCCT for suspected acute ischemic stroke. In our study, e-ASPECTS demonstrated strong diagnostic performance, with an accuracy of 96.3%, sensitivity of 96.9%, and specificity of 95.8%. These values are substantially higher than those reported in previous studies, where accuracy ranged from 67% to 87%, sensitivity from 14% to 83%, and specificity from 57 to 99% (Mallon et al., 2024; Austein et al., 2019; Nagel et al., 2017; Guberina et al., 2018). Many earlier studies focused exclusively on patients with confirmed anterior circulation acute ischemic stroke, and the ground truth was typically based on ASPECTS assessments performed by radiologists who were blinded to any automated results. In contrast, our cohort was broader and more representative of real-world clinical practice, including a wider range of patients encountered in routine stroke imaging. Perhaps the most appropriate comparison can be made with the study by Mallon et al., which evaluated the performance of the Brainomix e-Stroke software in a consecutive cohort of patients with suspected acute stroke, without excluding individuals based on clinical criteria. In that analysis, e-ASPECTS demonstrated an accuracy of 77%, a sensitivity of 57%, and a specificity of 84% (Mallon et al., 2024). These figures are notably lower than those observed in our study, prompting consideration of potential reasons for this discrepancy. One key difference is that in our setting, radiologists had access to the e-ASPECTS output during their manual ASPECTS evaluations. This may have influenced their assessments, potentially increasing concordance between manual and automated scores and leading to an overestimation of the software’s true performance.

Further support for this interpretation comes from the findings of Brinjikji et al., who investigated the impact of e-ASPECTS on interobserver agreement and diagnostic accuracy among a large group of radiologists and neurologists. Their results showed that access to the e-ASPECTS output significantly improved both consistency and accuracy (Brinjikji et al., 2021). This suggests that the enhanced performance observed in our study may, in part, reflect the beneficial influence of the software as a clinical decision support tool, rather than the standalone diagnostic accuracy of the algorithm.

While this study focused on overall and dichotomized ASPECTS analysis to reflect practical clinical decision-making, we recognize that this design differs from per-region validation approaches adopted in previous studies such as those by Nagel et al., Austein et al., and Guberina et al. Methodological differences between global and regional analyses likely contribute to the variation in reported diagnostic performance (Austein et al., 2019; Nagel et al., 2017; Nagel et al., 2018; Guberina et al., 2018).

A recent meta-analysis by Adamou et al. (2023) reported only moderate reliability between automated and expert ASPECTS readings, with a pooled intraclass correlation coefficient (ICC) of 0.54 (95% CI 0.40–0.67) (Adamou et al., 2023). In contrast, our study showed a strong positive correlation (*ρ* = 0.953, *p* < 0.001) and a Cohen’s *κ* of 0.88, indicating substantially higher concordance. Several factors may explain this discrepancy. First, radiologists in our cohort had access to the e-ASPECTS output during assessment, which likely enhanced alignment between automated and manual scoring, a scenario that reflects real-world clinical integration rather than blinded comparison. Second, our study was based on a single-center, standardized imaging workflow using modern scanners, minimizing technical variability that can reduce software reliability. Finally, previous meta-analytic data pooled heterogeneous studies differing in reference standards, imaging quality, and patient selection, which may have diluted overall agreement estimates.

One of the primary aims of this study was to evaluate the suitability of e-ASPECTS for identifying patients eligible for mechanical thrombectomy. While a one-point discrepancy in ASPECTS scoring among patients with high scores (Sarraj et al., 2023; Huo et al., 2023; Yoshimura et al., 2022) is unlikely to significantly affect treatment decisions or clinical outcomes, accuracy becomes critical in the lower score range. In particular, for patients with ASPECTS scores below 6, where treatment eligibility is often determined, the precision of e-ASPECTS is essential to avoid inappropriate inclusion or exclusion from potentially life-saving thrombectomy. In our study, relying solely on e-ASPECTS for treatment decisions would have resulted in misclassification in four patients: two would have been incorrectly excluded from mechanical thrombectomy, and two would have been incorrectly deemed eligible. Although this represents only 0.4% of the total assessed cohort, closely aligning with the 0.6% misclassification rate reported by Mallon et al. (2024), the impact is far more pronounced within the subgroup of patients with ASPECTS scores below 6, where the misclassification rate rises to 11.4%. This finding highlights the critical importance of accuracy in this lower score range, where treatment decisions are particularly sensitive to even small errors in assessment.

In light of the evolving criteria for mechanical thrombectomy eligibility, our findings should be interpreted within the broader context of landmark clinical trials. Earlier pivotal studies, such as ESCAPE and REVASCAT, helped to establish the conventional ASPECTS ≥6 threshold, primarily to avoid treating patients with large established infarcts (Goyal et al., 2015; Jovin et al., 2015). More recently, trials including SELECT2, ANGEL-ASPECT, and RESCUE-Japan LIMIT have challenged this paradigm, demonstrating that patients with lower ASPECTS values (Yang et al., 2022; Barber et al., 2000; Naylor et al., 2017) may still benefit significantly from thrombectomy (Sarraj et al., 2023; Huo et al., 2023; Yoshimura et al., 2022). In our cohort, the concordance between e-ASPECTS and radiologist assessment was high across strata, with an accuracy of 92.6% for patients with ASPECTS ≥6 and 87.9% for those with ASPECTS <6. Although the accuracy in patients with ASPECTS <6 was somewhat lower, this subgroup was small (33 patients), and therefore, the results should be interpreted with caution. Notably, the overall misclassification rate in our cohort (0.4%) was comparable to previously reported figures, and although misclassification rose to 11.4% in the ASPECTS <6 stratum, this aligns with the clinical challenge of decision-making in borderline cases. In our study, only eight patients had ASPECTS scores below 4, and no misclassifications were observed in this subgroup. However, given the limited sample size, larger and more targeted studies are needed to draw definitive conclusions about the reliability of e-ASPECTS in this critical range. Taken together, these findings suggest that automated ASPECTS scoring can provide valuable decision support in real-world settings, particularly as eligibility thresholds expand and the margin for error in low-score patients becomes increasingly critical.

Our study achieved a processing success rate of 91.9%, which aligns well with the broader discussion around the importance of balancing output suppression and reliability in automated stroke imaging tools. While slightly lower than the 96.5% reported by Mallon et al. (2024), our rate still exceeds many earlier reports (61%–89.5%) (Mair et al., 2023; Mair et al., 2022) and supports the notion that consistent, high-quality imaging protocols significantly contribute to successful processing outcomes. The high success rate in our cohort likely reflects the standardized imaging workflow at our center, which, although not explicitly optimized for Brainomix e-Stroke, still provides a stable imaging environment. This contrasts with earlier studies based on heterogeneous or historical multi-center data, where variability in image acquisition parameters may have contributed to lower processing success.

The findings of this study underscore the considerable clinical value of e-ASPECTS as a decision support tool in the acute stroke setting. Time is a critical factor in determining eligibility for mechanical thrombectomy, and with faster decision-making directly linked with the shorter time to recanalisation, tools that can help streamline and standardize this decision are crucial, especially in centers without 24/7 neuroradiology coverage. Nevertheless, we emphasize that the use of e-ASPECTS should remain complementary to expert interpretation rather than serve as a standalone determinant of care. In particular, caution is warranted in low ASPECTS ranges, where even minor inaccuracies can have outsized consequences. The discrepancies in e-ASPECTS and ASPECTS shown in this study underscore that clinicians must ensure that all imaging for patients being evaluated for time-sensitive interventions, such as mechanical thrombectomy, undergoes thorough review by a qualified radiologist. Automated analysis tools like e-ASPECTS can provide valuable support, but should not be used as the sole basis for treatment decisions.

This study has several important limitations. First, there is currently no universally accepted gold standard for assessing early ischemic changes on NCCT. Diffusion-weighted MRI (DWI) is more sensitive for detecting acute infarction and has been used as a reference standard in several prior evaluations of automated ASPECTS tools (Hoelter et al., 2020; Mokin et al., 2017; Labeyrie et al., 2012). However, our objective was to evaluate the clinical utility of e-ASPECTS as a decision-support tool in routine practice, where DWI is not always available in the hyperacute setting. For this reason, radiologist-assigned ASPECTS served as the reference standard, reflecting real-world clinical workflows in which treatment decisions are based primarily on NCCT interpretation by experts. While DWI-based validation studies provide important insights into the theoretical accuracy of automated scoring, NCCT-based validation, such as ours, better reflects the practical value of e-ASPECTS in guiding acute treatment decisions. Additionally, while per-region evaluation was beyond the scope of this clinically focused analysis, future studies should incorporate region-specific assessment to enable more direct comparison with prior validation work. Such analyses would provide a more detailed understanding of the spatial performance of e-ASPECTS. Moreover, the radiologists assessing ASPECTS were not blinded and had access to the e-ASPECTS scores, potentially introducing bias. Their primary responsibility was to make rapid treatment decisions, and prior studies suggest that access to automated results can increase concordance between manual and automated scoring (Brinjikji et al., 2021). This may have led to an overestimation of e-ASPECTS accuracy in our cohort. Future research with blinded assessments is necessary to determine the true diagnostic performance of e-ASPECTS. Additionally, the subgroup of patients with low ASPECTS scores was small; given the growing interest in extending thrombectomy eligibility to this population, larger studies focused on this group are crucial.

## Conclusion

e-ASPECTS demonstrated high accuracy (96.3%), sensitivity (95.8%), specificity (96.9%), and strong concordance with radiologist-assigned ASPECTS scores in a suspected acute stroke cohort, and these findings support its utility as a clinical decision support tool. However, its use should complement, not replace, expert radiological interpretation, especially in patients with low ASPECTS scores, where treatment decisions are most sensitive. Future research should focus on blinded, multicentre validation studies and larger cohorts of patients with low ASPECTS to better establish its reliability across diverse clinical settings.

## Data Availability

The data analyzed in this study is subject to the following licenses/restrictions: Patients private information is included in the datasets. Requests to access these datasets should be directed to Mateusz Dorochowicz, m.dorochowicz@outlook.com.

## References

[ref1] Adamou A. Beltsios E. T. Bania A. Gkana A. Kastrup A. Chatziioannou A. . (2023). Artificial intelligence-driven ASPECTS for the detection of early stroke changes in non-contrast CT: a systematic review and meta-analysis. J. Neurointerv. Surg. 15, e298–e304. doi: 10.1136/jnis-2022-019447, 36522179

[ref2] Alawieh A. Vargas J. Fargen K. M. Langley E. F. Starke R. M. De Leacy R. . (2019). Impact of procedure time on outcomes of thrombectomy for stroke. J. Am. Coll. Cardiol. 73, 879–890. doi: 10.1016/j.jacc.2018.11.052, 30819354

[ref3] Austein F. Wodarg F. Jürgensen N. Huhndorf M. Meyne J. Lindner T. . (2019). Automated versus manual imaging assessment of early ischemic changes in acute stroke: comparison of two software packages and expert consensus. Eur. Radiol. 29, 6285–6292. doi: 10.1007/s00330-019-06252-2, 31076862

[ref4] Bal S. Bhatia R. Menon B. K. Shobha N. Puetz V. Dzialowski I. . (2015). Time dependence of reliability of noncontrast computed tomography in comparison to computed tomography angiography source image in acute ischemic stroke. Int. J. Stroke 10, 55–60. doi: 10.1111/j.1747-4949.2012.00859.x, 22974504

[ref5] Barber P. A. Demchuk A. M. Zhang J. Buchan A. M. (2000). Validity and reliability of a quantitative computed tomography score in predicting outcome of hyperacute stroke before thrombolytic therapy. ASPECTS Study Group. Alberta Stroke Programme Early CT Score. Lancet 355, 1670–1674. doi: 10.1016/S0140-6736(00)02237-6, 10905241

[ref6] Brinjikji W. Abbasi M. Arnold C. Benson J. C. Braksick S. A. Campeau N. . (2021). E-ASPECTS software improves interobserver agreement and accuracy of interpretation of aspects score. Interv. Neuroradiol. 27, 781–787. doi: 10.1177/15910199211011861, 33853441 PMC8673896

[ref7] Farzin B. Fahed R. Guilbert F. Poppe A. Y. Daneault N. Durocher A. P. . (2016). Early CT changes in patients admitted for thrombectomy: intrarater and interrater agreement. Neurology 87, 249–256. doi: 10.1212/WNL.0000000000002860, 27316243 PMC4955274

[ref8] Goebel J. Stenzel E. Guberina N. Wanke I. Koehrmann M. Kleinschnitz C. . (2018). Automated ASPECT rating: comparison between the frontier ASPECT score software and the Brainomix software. Neuroradiology 60, 1267–1272. doi: 10.1007/s00234-018-2098-x, 30219935

[ref9] Goyal M. Demchuk A. M. Menon B. K. Eesa M. Rempel J. L. Thornton J. . (2015). Randomized assessment of rapid endovascular treatment of ischemic stroke. N. Engl. J. Med. 372, 1019–1030. doi: 10.1056/NEJMoa1414905, 25671798

[ref10] Guberina N. Dietrich U. Radbruch A. Goebel J. Deuschl C. Ringelstein A. . (2018). Detection of early infarction signs with machine learning-based diagnosis by means of the Alberta Stroke Program Early CT Score (ASPECTS) in the clinical routine. Neuroradiology 60, 889–901. doi: 10.1007/s00234-018-2066-5, 30066278

[ref11] Heit J. J. Mlynash M. Christensen S. Kemp S. M. Lansberg M. G. Marks M. P. . (2021). What predicts poor outcome after successful thrombectomy in late time windows? J Neurointerv Surg. 13, 421–425. doi: 10.1136/neurintsurg-2020-016125, 32554693

[ref12] Hoelter P. Muehlen I. Goelitz P. Beuscher V. Schwab S. Doerfler A. (2020). Automated ASPECT scoring in acute ischemic stroke: comparison of three software tools. Neuroradiology 62, 1231–1238. doi: 10.1007/s00234-020-02439-3, 32382795

[ref13] Huo X. Ma G. Tong X. Zhang X. Pan Y. Nguyen T. N. . (2023). Trial of endovascular therapy for acute ischemic stroke with large infarct. N. Engl. J. Med. 388, 1272–1283. doi: 10.1056/NEJMoa2213379, 36762852

[ref14] Jovin T. G. Chamorro A. Cobo E. de Miquel M. A. Molina C. A. Rovira A. . (2015). Thrombectomy within 8 hours after symptom onset in ischemic stroke. N. Engl. J. Med. 372, 2296–2306. doi: 10.1056/NEJMoa1503780, 25882510

[ref15] Labeyrie M.-A. Turc G. Hess A. Hervo P. Mas J.-L. Meder J.-F. . (2012). Diffusion lesion reversal after thrombolysis: a MR correlate of early neurological improvement. Stroke 43, 2986–2991. doi: 10.1161/STROKEAHA.112.661009, 22996954

[ref16] Mainali S. Wahba M. Elijovich L. (2014). Detection of early ischemic changes in noncontrast CT head improved with “stroke windows”. ISRN Neurosci. 2014:654980. doi: 10.1155/2014/654980, 24967315 PMC4045559

[ref17] Mair G. White P. Bath P. M. Muir K. W. Al-Shahi Salman R. Martin C. . (2022). External validation of e-ASPECTS software for interpreting brain CT in stroke. Ann. Neurol. 92, 943–957. doi: 10.1002/ana.26495, 36053916 PMC9826303

[ref18] Mair G. White P. Bath P. M. Muir K. Martin C. Dye D. . (2023). Accuracy of artificial intelligence software for CT angiography in stroke. Ann. Clin. Transl. Neurol. 10, 1072–1082. doi: 10.1002/acn3.51790, 37208850 PMC10351662

[ref19] Mallon D. Fallon M. Blana E. McNamara C. Menon A. Ip C. L. . (2024). Real-world evaluation of Brainomix e-stroke software. Stroke Vasc. Neurol. 9, 497–504. doi: 10.1136/svn-2023-002859, 38164621 PMC11732836

[ref20] Mokin M. Primiani C. T. Siddiqui A. H. Turk A. S. (2017). ASPECTS (Alberta stroke program early CT score) measurement using Hounsfield unit values when selecting patients for stroke thrombectomy. Stroke 48, 1574–1579. doi: 10.1161/STROKEAHA.117.016745, 28487329

[ref21] Mueller F. Fabritius M. P. Stueckelschweiger L. Kiesl S. Moench S. Tiedt S. . (2022). CT after interhospital transfer in acute ischemic stroke: imaging findings and impact of prior intravenous contrast administration. Front. Neurol. 13:1023147. doi: 10.3389/fneur.2022.1023147, 36570440 PMC9767970

[ref22] Nagel S. Sinha D. Day D. Reith W. Chapot R. Papanagiotou P. . (2017). E-ASPECTS software is non-inferior to neuroradiologists in applying the ASPECT score to computed tomography scans of acute ischemic stroke patients. Int. J. Stroke 12, 615–622. doi: 10.1177/1747493016681020, 27899743

[ref23] Nagel S. Wang X. Carcel C. Robinson T. Lindley R. I. Chalmers J. . (2018). Clinical utility of electronic Alberta stroke program early computed tomography score software in the ENCHANTED trial database. Stroke 49, 1407–1411. doi: 10.1161/STROKEAHA.117.019863, 29777016

[ref24] Naylor J. Churilov L. Rane N. Chen Z. Campbell B. C. V. Yan B. (2017). Reliability and utility of the Alberta stroke program early computed tomography score in hyperacute stroke. J. Stroke Cerebrovasc. Dis. 26, 2547–2552. doi: 10.1016/j.jstrokecerebrovasdis.2017.05.042, 28652060

[ref25] Neuhaus A. Seyedsaadat S. M. Mihal D. Benson J. C. Mark I. Kallmes D. F. . (2020). Region-specific agreement in ASPECTS estimation between neuroradiologists and e-ASPECTS software. J. Neurointerv. Surg. 12, 720–724. doi: 10.1136/neurintsurg-2019-015442, 31818971

[ref26] Nicholson P. Hilditch C. A. Neuhaus A. Seyedsaadat S. M. Benson J. C. Mark I. . (2020). Per-region interobserver agreement of Alberta stroke program early CT scores (ASPECTS). J. Neurointerv. Surg. 12, 1069–1071. doi: 10.1136/neurintsurg-2019-015473, 32024784

[ref27] Pfaff J. Herweh C. Schieber S. Schönenberger S. Bösel J. Ringleb P. A. . (2017). E-ASPECTS correlates with and is predictive of outcome after mechanical thrombectomy. AJNR Am. J. Neuroradiol. 38, 1594–1599. doi: 10.3174/ajnr.A5236, 28596195 PMC7960437

[ref30] R Core Team (2021) R: A language and environment for statistical computing. Vienna, Austria: R Foundation for Statistical Computing.

[ref28] Samaniego E. A. Boltze J. Lyden P. D. Hill M. D. Campbell B. C. V. Silva G. S. . (2023). Priorities for advancements in neuroimaging in the diagnostic workup of acute stroke. Stroke 54, 3190–3201. doi: 10.1161/STROKEAHA.123.044985, 37942645 PMC10841844

[ref29] Sarraj A. Hassan A. E. Abraham M. G. Ortega-Gutierrez S. Kasner S. E. Hussain M. S. . (2023). Trial of endovascular thrombectomy for large ischemic strokes. N. Engl. J. Med. 388, 1259–1271. doi: 10.1056/NEJMoa2214403, 36762865

[ref31] Yang S. Yao W. Siegler J. E. Mofatteh M. Wellington J. Wu J. . (2022). Shortening door-to-puncture time and improving patient outcome with workflow optimization in patients with acute ischemic stroke associated with large vessel occlusion. BMC Emerg. Med. 22:136. doi: 10.1186/s12873-022-00692-8, 35883030 PMC9315077

[ref32] Yoshimura S. Sakai N. Yamagami H. Uchida K. Beppu M. Toyoda K. . (2022). Endovascular therapy for acute stroke with a large ischemic region. N. Engl. J. Med. 386, 1303–1313. doi: 10.1056/NEJMoa2118191 35138767

[ref33] Yu A. C. Mohajer B. Eng J. (2022). External validation of deep learning algorithms for radiologic diagnosis: a systematic review. Radiol. Artif. Intell. 4:e210064. doi: 10.1148/ryai.210064 35652114 PMC9152694

